# The Evaluation Value of Diffusion-Weighted Imaging for Brain Injury in Patients after Deep Hypothermic Circulatory Arrest

**DOI:** 10.1155/2022/5985806

**Published:** 2022-05-26

**Authors:** Jiaxiang Zhuang, Xiandong Lin, Jianbing Lin, Shun Yu, Shuangbo Dai, Licheng Yan, Yuanxiang Chen, Ren Wang

**Affiliations:** ^1^Department of Cardiovascular Surgery, Shengli Clinical College of Fujian Medical University, Fujian Provincial Hospital, Fuzhou 350001, China; ^2^Department of Anesthesiology, Shengli Clinical College of Fujian Medical University, Fujian Provincial Hospital, Fuzhou 350001, China; ^3^Radiology Department, Shengli Clinical College of Fujian Medical University, Fujian Provincial Hospital, Fuzhou 350001, China

## Abstract

**Objective:**

Cerebral complications may occur after surgery with deep hypothermic circulatory arrest (DHCA). Diffusion-weighted imaging (DWI) has shown promising results in detecting early changes of cerebral ischemia. However, studies in human models are limited. Here, we examined the significance of DWI for detecting brain injury in postoperative patients after DHCA.

**Methods:**

Twelve patients who had undergone selective cerebral perfusion with DHCA were enrolled. All patients underwent magnetic resonance imaging (MRI) examinations before and after the operation with T1-weighted phase (T1W) and T2-weighted phase (T2W). Magnetic resonance angiography (3D TOF) was applied to observe intracranial arterial communication situations. DWI was employed to calculate the apparent diffusion coefficient (ADC) values. The neurocognitive function of patients was assessed preoperatively and postoperatively using the Montreal Cognitive Assessment Scale (MoCA), Hamilton Depression Scale (HAMD), and Hamilton Anxiety Scale (HAMA).

**Results:**

The ADC values of the whole brain of patients after surgery were significantly higher than before surgery (*P* = 0.003). However, no significant difference in the ADC values of other regions before and after the operation was observed. There was no significant effect on the postoperative cognitive function of patients after surgery, but visual-spatial and executive abilities were significantly reduced, while psychological anxiety (*P* = 0.005) and depression levels (*P* < 0.05) significantly increased. Correlation analysis revealed a significant association between ADC change values and depression change values (*P* < 0.05).

**Conclusion:**

DHCA demonstrated no significant effect on the cognitive function of patients but could affect the mood of patients. On the other hand, DWI demonstrated promising efficiency and accuracy in evaluating brain injury after DHCA.

## 1. Introduction

The central nervous system (CNS) consists of the brain and the spinal cord, which are highly sensitive to external mechanical injury and damage [1]. The CNS usually exhibits limited resilience, especially regarding synaptic network reconstruction after injury [2], partly due to its inability to restore damaged neurons or synaptic networks at the injured site [3]. The CNS can suffer from different types of external injuries that affect its function and structure, including stroke, spinal cord injury, and traumatic brain injury [4]. CNS damage has been observed to affect millions of people each year and is one of the most common causes of death or disability in patients [5, 6], and can at times be fatal due to the limited effects of currently available treatments [7].

In addition, CNS injury has been the most prominent complication and fatal factor in cardiac surgery [8]. Deep hypothermic circulatory arrest (DHCA) technology is widely used in complex congenital heart disease, aortic arch, and thoracic aortic surgery due to the need of various complex and difficult cardiovascular procedures [9]. However, the ability of the CNS to tolerate ischemia limits the safe time limit of circulatory arrest. Although advances in surgical techniques, anesthesia management, extracorporeal circulation techniques, and postoperative monitoring have been achieved over the past 20 years, postoperative stroke and other neurological complications remain prominent [10].

Postoperative cognitive dysfunction (POCD) is a common neurological complication after cardiac surgery [11]. The high incidence of POCD greatly impacts patients' postoperative recovery and quality of life, often leading to increased mortality, delayed rehabilitation, increased other complications, and even permanent cognitive impairment [12]. Early detection of brain injuries is essential for the timely treatment of patients and reducing the risks of CNS complications. Although many studies have shown that DHCA is a safe and reliable technique [13, 14], many negative effects have also been found in recent years [15–17].

Diffusion-weighted imaging (DWI) is an emerging neuroimaging technique that reflects the diffusion motion of water molecules based on the echo-planar imaging (EPI) technique [15, 18]. The application of DWI in neurogenic diseases was reported as early as 1985 [19]. Since then, DWI techniques have been mainly used to diagnose acute cerebral infarction and intracranial tumors [20]. DWI differs from traditional MRI techniques as its signal is derived from intrinsic tissue contrast and therefore does not require an injection of contrast agents as it mainly relies on the movement of water molecules rather than spin-proton density imaging [16, 21]. The diffusion of water molecules in tissues is affected by various factors, such as cell structure and biochemical characteristics of tissues, which can change the distance and direction of diffusion [15, 17]. DWI uses gradient fields sensitive to the path and direction of diffusion to show the diffusion of water molecules in the human body [22]. After the occurrence of ischemia, the diffusion of water molecules in the local brain tissue is usually limited to a certain extent. DWI has been shown to be sensitive to the early changes of cerebral ischemia and can detect lesions earlier than conventional magnetic resonance detection [23]. Further, Wang et al. showed that DW1 was superior to T1WI or T2WI in detecting early neurological changes in pigs after DHCA [24]. However, similar observations in human models are limited. Further, considering that there have been several controversies regarding DHCA, its clinical impact on patients' cognitive function should be re-evaluated.

In this study, we investigated the effects of DHCA on the cognitive function of patients after surgery and used DWI to evaluate the efficacy and safety of the DHCA for detecting brain injury in postoperative patients.

## 2. Materials and Methods

### 2.1. Study Subjects

Twelve patients who underwent selective cerebral perfusion with DHCA after admission from February 2019 to September 2021 were selected for this study. The inclusion criteria were: (1) Patients who are aged 20–60 years old; (2) Education levels above primary school with no communication barriers; (3) Patients who received magnetic resonance imaging and neurophysiological function tests; (4) Patients with chronic type A dissection (an onset time is more than 2 weeks), aortic arch aneurysm, or arch dissection; (5) Patients with dissection reversed to the aortic arch or ascending aorta after implantation of B-type dissection stent-graft, proximal unblockable thoracic patients with abdominal aortic aneurysm or dissection; (6) Patients who participated voluntarily and provided signed informed consent. Patients were excluded if they: (1) dropped out of the experiment halfway or succumbed to the surgery; (2) could not complete preoperative and postoperative neurophysiological tests; (3) did not receive diffusion-weighted magnetic resonance examinations after surgery; (4) had anomalies in the 3 items of thyroid function, folic acid + vitamin B12, and oxygen partial pressure indicators. The gender, age, educational level, clinical diagnosis, surgical method, cardiopulmonary bypass time, aortic cross-clamp time, and circulatory arrest selective cerebral perfusion time were recorded.

The study protocol was approved by the ethics committee of the Shengli Clinical College of Fujian Medical University, Fujian Provincial Hospital (Fuzhou, China). All patients provided written informed consent before participating, and this study fully adheres to the Declaration of Helsinki and other bioethical principles.

### 2.2. Treatment with DHCA

An arterial perfusion tube was inserted through the axillary artery. A two-step venous drainage tube was inserted into the right atrium to establish extracorporeal circulation. A left heart drainage tube was inserted into the right upper pulmonary artery, followed by cooling. A proximal aorta operation was performed while cooling. When the nasopharyngeal temperature dropped to 20°C, lower body circulation was stopped, and the innominate artery, left common carotid artery, and left subclavian artery were blocked. The extracorporeal circulation flow was reduced from full flow to 10 mL/kg/min, and selective cerebral perfusion was performed via axillary artery cannulation. Intraoperative stent implantation was performed, followed by anastomosis of the four branch vessels with the stent vessels and autologous aorta. After the distal aortic anastomosis was completed, another arterial perfusion tube was inserted through the 10 mm branch vessel of the 4 bifurcated vessels. A single pump double tube was used to restore full flow and lower body circulation. DHCA was used if the proximal descending aorta could not be blocked for patients undergoing thoracoabdominal aortic replacement through a combined thoracoabdominal incision. Extracorporeal circulation was established through femoral arteriovenous catheterization, and left ventricular drainage tubes were inserted through the left apex and pulmonary artery. The temperature was then lowered. When the nasopharyngeal temperature dropped to 20°C, the flow rate was reduced to two-thirds of the full flow rate, the descending aorta was blocked, circulation in the upper body was stopped, and the left heart was drained through the left heart drainage tube. An open anastomosis of the quadrifurcation vessel and the proximal descending aorta was performed. After the proximal anastomosis of the descending aorta was completed, another arterial perfusion tube was inserted through the 10 mm branch vessel of the artificial quadrifurcation blood vessel, and a single pump double tube was used to restore full flow and upper body circulation.

### 2.3. Magnetic Resonance Testing

Cranial MRI was performed on the patients before and after surgery to acquire T1-weighted (T1W), T2-weighted (T2W), and DWI images. DWI sequences were postprocessed on a GE workstation ADW4.2 using the Functool software. For each DWI level, apparent diffusion coefficient (ADC) values were calculated from 4 slices: cerebellum, basal ganglia, occipital lobe, and parietal lobe. In addition, magnetic resonance angiography (3D time-of-flight, 3D TOF) was used for carotid and intracranial arterial imaging to observe the intracranial arterial communication of the patients.

### 2.4. Cognitive Psychological Assessment

All patients were evaluated by the Montreal Cognitive Assessment (MoCA) [25], the Hamilton Depression Scale (HAMD) [26], and the Hamilton Anxiety Scale (HAMA) [27] before and after the surgery for neurocognitive function assessment. The postoperative neurocognitive function assessment time was consistent with the head magnetic resonance examination time.

### 2.5. Statistical Analysis

The SPSS 26.0 software was used to analyze the data of this study. Continuous variables were described by the mean ± standard deviation (Mean ± SD), and *T*-test analysis was used to compare the two groups. *P* < 0.05 was used as the criterion to judge the significance of the differences observed between groups.

## 3. Results

### 3.1. Patient Clinical Characteristics

Twelve patients, comprising of 8 males and 4 females, were eligible for this study. The mean age was 38 years and had an education level of primary school or above. All patients were diagnosed with aortic dissection. The main surgical methods were Bentall + Sun, with an extracorporeal circulation time greater than 120 minutes, an aortic cross-clamp time greater than 75 minutes, and circulatory arrest selective cerebral perfusion time greater than 10 minutes (Table 1).

### 3.2. The Effects of DHCA on the Cognitive and Psychological Functions of Patients

First, the effects of DHCA on patients' cognitive and psychological function were evaluated. The results showed that the visual-spatial and executive abilities of the patients after surgery were significantly lower than those before surgery (*P* = 0.002). There was no significant difference in naming ability, attention, language ability, abstract ability, delay ability, orientation ability, and total score. According to the statistical results of the postoperative orientation (*P* = 0.005) and total score (*P* = 0.002), the patients' score were significantly reduced after surgery, but there was no significant difference in repetition ability, mental arithmetic ability, recall ability, recognition ability, language ability, execution ability, and drawing ability (Table 2). According to the statistical results of the HAMD and HAMA (Table 2), the postoperative psychological anxiety (*P* = 0.005), somatic anxiety (*P* = 0.005), body weight score (*P* = 0.002), cognitive impairment (*P* = 0.002), sleep disorders (*P* = 0.003), total anxiety score (*P* = 0.002), and total depression score (*P* = 0.002) of patients were significantly increased, and the grade of anxiety (*P* = 0.028) and depression (*P* ≤ 0.001) were significantly increased. These data suggest that DHCA had no significant effect on cognitive function but could be associated with anxiety and depression.

### 3.3. DWI Detection and ADC Value Comparison of Each Plane of the Brain before and after Surgery

**Figure 1:**
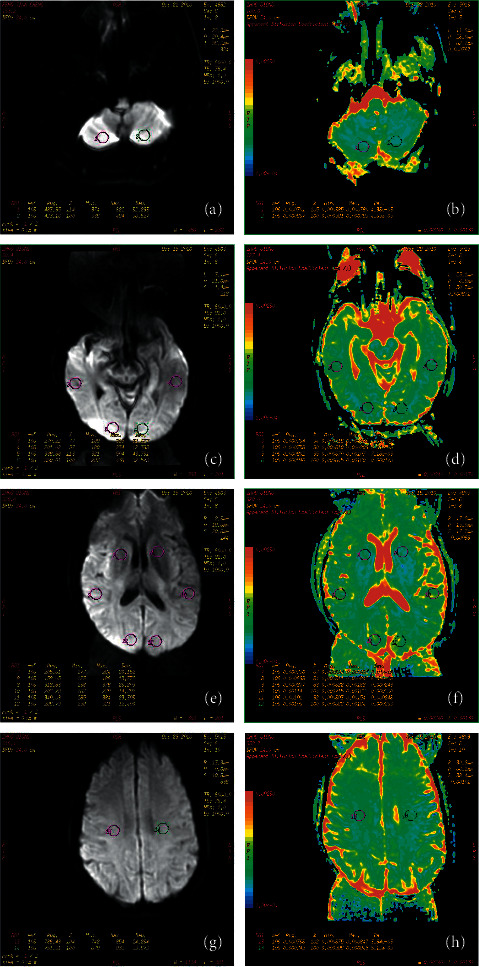
Diffusion-weighted imaging of each plane of the brain before surgery (a) Plane of cerebellum DWI (b) Plane of cerebellum ADC (c) Basal ganglia plane DWI (d) Basal ganglia plane ADC (e) Occipital plane DWI (f) Occipital plane ADC (g) Parietal plane DWI (h) Parietal plane ADC.

By analyzing the results of magnetic resonance examination before and after surgery, we observed that 9 patients had anterior and posterior cerebral artery communication; 1 patient lacked the right posterior communication; 1 patient lacked the bilateral posterior communication; and 1 patient had no connection between the left anterior and middle cerebral arteries after surgery. The ADC values in different planes of the patients' brain were further calculated based on DWI (Figure 1(a)–1(h)). The results showed that the ADC values of different brain planes were different. Further assessment showed that the ADC value of the whole brain level of the patients after surgery was significantly higher than before surgery (*P* = 0.003). However, there was no significant difference in ADC values at five cerebellum levels, basal ganglia, occipital lobe, and parietal lobe before and after surgery. In addition, there was no significant difference in ADC values between the left and right brains of patients before and after surgery (Table 3).

### 3.4. Correlation Analysis between ADC Value and Neuropsychological Function Test

**Figure 2:**
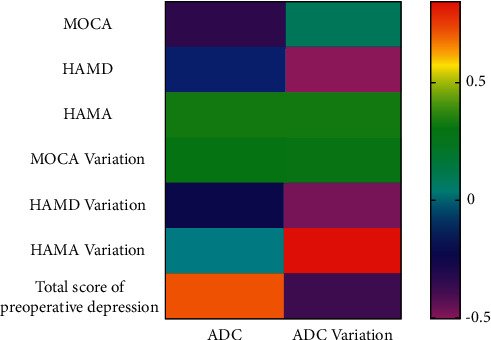
Correlation analysis between ADC values and neuropsychological function.

The correlation between ADC values and neuropsychological function scores was subsequently investigated. The results showed no correlation between the ADC value change and the anxiety value change or MoCA total score value change. Still, ADC value changes were significantly correlated with HAMA scores (*P* < 0.05; Figure 2), suggesting that the ADC values are related to patient depression levels.

## 4. Discussion

To understand the patients' specific situation, DWI was used, and it demonstrated promising abilities to accurately diagnose the anterior and posterior communication of the cerebral arteries and clearly show the communication sites. This could be related to diffusion-weighted magnetic resonance imaging, a quantitative technique that exploits the diffusion of water within biological tissues [28]. The ADC is a value that measures the ease of this translational motion of water and can be used to reflect the degree of destruction of tissue architecture due to injuries [13, 14]. In biological tissues, this coefficient is lower than that of free water due to the various structures of the tissue hindering the free movement of water molecules [29]. Several currently known CNS pathological conditions are associated with significant changes in the diffusion properties of associated tissues. For example, pathological processes that compromise tissue integrity often reduce barriers to water movement, and these processes tend to increase measured ADC values [30]. At the same time, Burke et al. demonstrated that DWI has a high sensitivity (88%–100%) in detecting acute ischemia, compared with computed tomography [31], indicating that DWI imaging could help in better judging patients' blood flow. This study also detected that 9 patients had anterior and posterior cerebral artery communication; 1 patient lacked right posterior communication; 1 patient lacked bilateral posterior communication; and 1 patient had no connection between the left anterior and middle cerebral arteries after surgery.

DWI has repeatedly been shown to be the most sensitive technique for identifying acute ischemia because of its ability to detect rapid changes in the ratio of extracellular to intracellular water content in the brain [32]. When tissue ischemia and hypoxia occur, energy metabolism disorder occurs in brain cell hypoxia in the acute phase [33]. This directly inhibits the activity of sodium/potassium ATPase on the plasma membrane, resulting in a significant outflow of potassium ions and an influx of calcium ions, chloride ions, and sodium ions that accumulate in cells forming an intracellular hypertonic state. Many water molecules then enter, causing cytotoxic edema and resulting in an ADC value that should be reduced [33]. However, our results showed that the ADC value of the whole brain was increased, which may be due to the limitation of clinical conditions. We performed postoperative magnetic resonance imaging quite late (4–7 days after surgery), and the acute phase of the injury could have already occurred by that time. In the absence of severe injury, the cytotoxic edema subsided. Due to cell lysis, the diffusion of water molecules is enhanced in some necrotic cells, and the ADC value increases [34].

At present, DHCA has been widely used as a cerebral protective strategy in clinical practice [35]. Studies have shown that DHCA can reduce cerebral metabolism rates compared with other traditional methods, release free radicals in neuronal tissue tissues, and avoid postischemia and cerebral edema [36, 37]. At the same time, the adverse effects of DHCA should not be ignored [38]. The study of Chau et al. pointed that compared with the non-DHCA treatment group, the cognitive function, processing speed, and executive function of the DHCA treatment group were significantly improved but possessed specific impairments [39]. This study also found that postoperative visual-spatial and executive abilities were significantly reduced, while anxiety and depression levels were significantly increased. At the same time, studies have also reported the adverse effects of patients' experiences could be due to the disruption of the metabolic properties of the hippocampus by DHCA [17]. The hippocampus plays a crucial role in memory formation, but it has a high metabolic rate and is therefore particularly sensitive to ischemic injury and highly vulnerable to disruption [40]. These results also suggest that DHCA technology can effectively reduce the impairment of cognitive brain function caused by aortic surgery. Still, it needs to be used simultaneously as the early-stage judgment of nerve injury in patients.

The correlation analyses of this study showed that the ADC value changes were not correlated with anxiety value changes, MoCA total score value changes, or MoCA total scores. These observations could be because DHCA technology could avoid damage to patients' cerebral cortex and hindbrain, thereby protecting patients' naming ability, attention, language, abstract ability, delayed recall, orientation ability, etc. [41]. However, ADC was associated with changes in depression values in patients, indicating that patients experienced emotional fluctuations before and after surgery, which may be due to the effects of DHCA on the hippocampus [42].

## 5. Conclusion

In conclusion, our results showed that DWI was sensitive in detecting and evaluating brain injury after DHCA. Although DHCA did not significantly affect the cognitive functions of patients after surgery, it had some effects on the visual-spatial execution and emotions of the patients. These findings indicate that DHCA could be clinically safe, but due to the small sample size and short follow-up of this study, our findings should be confirmed in larger sample size and long-term follow-up studies.

## Figures and Tables

**Table 1:** Patients' general data.

Serial number	Gender	Age	Education level	Clinical diagnosis	Surgical method	Extracorporeal circulation time	Aortic cross-clamp time	Circulatory arrest selective cerebral perfusion time
1	Male	44	High school	Aortic dissection A3C	Bentall + Sum	164	76	24
2	Male	37	Junior high	Aortic dissection A2C, hypertension level 3	Bentall + Sum	223	137	31
3	Male	54	High school	Aortic dissection A3C	Bentall + Sum	196	105	16
4	Male	25	Junior high	Marfan syndrome, aortic dissection A3C	Bentall + Sum	179	96	13
5	Female	28	University	Aortic dissection A3C	Bentall + Sum	204	124	29
6	Male	19	College	Aortic dissection A3C	Bentall + Sum	200	86	19
7	Male	32	College	Retrograde aortic dissection after descending aorta and right renal artery stent implantation	Bentall + Sum	192	80	26
8	Male	49	High school	Aortic dissection A3C	Bentall + Sum	171	75	18
9	Female	43	University	Retrograde dissection after descending aortic stent implantation	Aortic valvuloplasty + ascending aortic replacement + Sum	154	78	18
10	Female	31	Primary school	Ascending aortic aneurysm, Takayasu arteritis, bilateral carotid artery occlusion	Bentall + partial arch + ascending aorta to double carotid artery bypass	186	90	10
11	Male	62	College	Aortic dissection	Ascending aorta + Sum	148	90	19
12	Female	36	University	Aortic dissection A2C	Ascending aortic replacement + Sum + CABG	304	214	45

**Table 2:** Comparison of neuropsychological function scores before and after surgery.

Neuropsychological function		Before surgery	After surgery	*Z*	*P*
MoCA	Visual-spatial and executive ability	4 ± 1	2 ± 1	3.035	0.002^*∗∗*^
	Naming ability	2 ± 0	2 ± 0	1.000	0.317
	Attention	4 ± 1	4 ± 1	0.000	1.000
	Language	2 ± 0	2 ± 1	1.414	0.157
	Abstract	4 ± 1.25	4.5 ± 1	1.633	0.102
	Orientation	5 ± 0.25	5 ± 0.25	0.000	1.000
	Total score	21 ± 2.5	20.5 ± 2	1.496	0.135

HAMD	Somatic anxiety	4 ± 2.5	7 ± 6.25	2.796	0.005^*∗∗*^
	Psychological anxiety	5.5 ± 5.25	12 ± 4.75	2.828	0.005^*∗∗*^
	Anxiety total score	9 ± 4.75	19 ± 8	2.938	0.002 ^*∗∗*^
	Anxiety Grade(Yes/No)	8/4	12/0	4.800	0.028^*∗*^

HAMA	Anxious somatization	2 ± 2.25	6 ± 2.25	3.068	0.002^*∗∗*^
	Weight	0 ± 2	6 ± 1.5	3.068	0.002^*∗∗*^
	Cognitive impairment	0 ± 0	6 ± 2.25	3.074	0.002^*∗∗*^
	Sluggish	0.5 ± 1	0 ± 1	1.000	0.317
	Sleeping disorder	0 ± 1.25	7.5 ± 2.25	2.991	0.003^*∗∗*^
	Depression total score	4 ± 5.5	23.5 ± 6.5	3.061	0.002^*∗∗*^
	Depression Grade(Yes/No)	3/9	12/0	14.400	≤0.001^*∗∗∗*^

*Note.* For depression and anxiety, a score of >7 points was counted, including mild, moderate, and severe cases; *P* < 0.01, *P* < 0.001 vs. Preoperative.

**Table 3:** Comparison of ADC values before and after surgery.

Scanning position	Before surgery	After surgery	*t*	*P*
Whole brain	8.99 ± 2.54	11.15 ± 2.06	3.728	0.003 ^*∗∗*^
Cerebellum	8.19 ± 0.72	8.14 ± 0.84	0.366	0.721
Basal ganglia	8.25 ± 0.49	8.24 ± 0.45	0.235	0.818
Occipital lobe	8.20 ± 0.67	8.25 ± 0.53	0.653	0.527
Parietal lobe	8.58 ± 0.94	8.60 ± 0.67	0.234	0.819
Left brain	8.15 ± 0.72	8.21 ± 0.60	0.926	0.374
Right brain	8.63 ± 0.92	8.58 ± 0.81	0.550	0.593

^*∗∗*^ *P* < 0.01 vs. Before Surgery.

## Data Availability

The data used to support the findings of this study are available from the corresponding author upon request.
